# Class 1C antiarrhythmic drugs in atrial fibrillation and coronary artery disease

**DOI:** 10.1111/jce.14335

**Published:** 2020-01-24

**Authors:** Peter G. Pantlin, Robert M. Bober, Michael L. Bernard, Sammy Khatib, Glenn M. Polin, Paul A. Rogers, Daniel P. Morin

**Affiliations:** ^1^ Department of Cardiology, Ochsner Clinical School University of Queensland New Orleans Louisiana; ^2^ Department of Cardiology Ochsner Medical Center New Orleans Louisiana

**Keywords:** adverse drug effect, atrial fibrillation, coronary artery disease, drug therapy, 1C antiarrhythmic drugs, ischemic heart disease

## Abstract

**Background:**

Class 1C antiarrhythmic drugs (AADs) are effective first‐line agents for atrial fibrillation (AF) treatment. However, these agents commonly are avoided in patients with known coronary artery disease (CAD), due to known increased risk in the postmyocardial infarction population. Whether 1C AADs are safe in patients with CAD but without clinical ischemia or infarct is unknown. Reduced coronary flow capacity (CFC) on positron emission tomography (PET) reliably identifies myocardial regions supplied by vessels with CAD causing flow limitation.

**Objective:**

To assess whether treatment with 1C AADs increases mortality in patients without known CAD but with CFC indicating significantly reduced coronary blood flow.

**Methods:**

In this pilot study, we compared patients with AF and left ventricular ejection fraction ≥50% who were treated with 1C AADs to age‐matched AF patients without 1C AAD treatment. No patient had clinically evident CAD (ie, reversible perfusion defect, known ≥70% epicardial lesion, percutaneous coronary intervention, coronary artery bypass grafting, or myocardial infarction). All patients had PET‐based quantification of stress myocardial blood flow and CFC. Death was assessed by clinical follow‐up and social security death index search.

**Results:**

A total of 78 patients with 1C AAD exposure were matched to 78 controls. Over a mean follow‐up of 2.0 years, the groups had similar survival (*P* = .54). Among patients with CFC indicating the presence of occult CAD (ie, reduced CFC involving ≥50% of myocardium), 1C‐treated patients had survival similar to (*P* = .44) those not treated with 1C agents.

**Conclusions:**

In a limited population of AF patients with preserved left ventricle function and PET‐CFC indicating occult CAD, treatment with 1C AADs appears not to increase mortality. A larger study would be required to confidently assess the safety of these drugs in this context.

## 1 INTRODUCTION

Atrial fibrillation (AF) is the most common abnormal heart rhythm in the United States, with a prevalence of approximately 6.1 million people.^1^ Class 1C antiarrhythmic drugs (AADs), such as flecainide and propafenone, are first‐line agents for AF in the current ACC/AHA/HRS guidelines.^2^ However, the cardiac arrhythmia suppression trial (CAST) showed that following myocardial infarction (MI), the use of 1C AADs, while effective for premature ventricular contraction (PVC) suppression, was associated with increased mortality and nonfatal cardiac arrest.^3^ The proarrhythmic effect of 1C drugs likely is related to their effect of decreasing myocardial conduction velocity, which predisposes to myocardial reentry around and through regions of infarcted myocardium.^3^ In addition, some evidence links adverse events with an interplay between ongoing ischemia and class 1C AAD use.^4^

Due to an extension of the CAST results, which were reported three decades ago, many clinicians still commonly avoid AF treatment with 1C agents in patients with any known coronary artery disease (CAD), due to perceived increased risk.^5^ Whether the existence of CAD but without infarction or clinically evident ischemia truly increases the risk of 1C AAD use is unknown.

Positron emission tomography (PET) can quantify coronary flow capacity (CFC), which is a novel metric defined by the integration of stress myocardial blood flow (sMBF) and coronary flow reserve into a framework of physiologic severity.^6^ Mild reduction in CFC dependably identifies myocardial regions supplied by vessels with CAD but without clinical evidence of ischemia or infarction.^7^, ^8^, ^9^ Thus, PET‐based assessment of coronary flow is able to detect patients who have the presence of CAD that may not be clinically evident otherwise. Some patients without clinical CAD but with reduced CFC are treated with 1C AADs. This represents a unique natural experiment, allowing comparison of survival between patients with existent but nonevident CAD who was treated with 1C AADs, and similar patients who were not exposed to 1C AADs.

## 2 METHODS

This retrospective cohort study was approved by the Ochsner Medical Center Institutional Review Board. We included patients with AF and without clinical CAD who had undergone a cardiac PET scan that assessed absolute myocardial blood flow and CFC. All therapy, including general and antiarrhythmic medical therapy, was at the discretion of the treating physician. Typical angina was defined as chest pain on exertion. The clinically evident CAD was defined as any history of the acute coronary syndrome, coronary revascularization, MI, significant epicardial arterial stenosis, or relative perfusion abnormality on PET imaging. A relative perfusion abnormality indicating overt CAD was defined as a contiguous defect in greater than 10% of the left ventricle (LV) myocardium with activity less than 60% of maximum at rest, or a contiguous defect in greater than 5% of LV myocardium with activity less than 60% of maximum at stress. The occult CAD was defined as the absence of evidence of overt CAD but with the presence of reduced CFC involving ≥50% of the myocardium.^6^, ^7^

Sudden cardiac death was defined as a sudden unexpected pulseless condition due to a likely cardiac etiology if observed; or death within 24 hours of last being seen in the usual state of health if not observed. Nonsudden cardiac death was defined as death due to a cardiac cause that was not arrhythmic (eg, progressive heart failure). Adjudication of these events was accomplished via chart review.

We used our electronic medical record to identify all patients who had AF and had undergone PET stress testing. Patients with clinically known CAD, perfusion abnormalities on PET, or left ventricular ejection fraction (LVEF) less than 50% on echocardiography were excluded. Within this group, we identified patients who were treated with 1C AADs either at the time of PET scanning or were initiated with 1C AADs within 1 month thereafter. We then assembled the control cohort, composed of one “no‐1C AAD” patient for each patient in the 1C AAD cohort, matched as closely as possible based on age. Investigators were blinded to all other data during this process.

The electronic medical record was used to obtain demographic data (age, sex, chronic medical problems, and medications) and clinical data (blood pressure, body mass index, and laboratory data) at the time of PET scanning. All patients had PET‐based quantification of sMBF and CFC. At the time of this study, CFC data were not shared with the ordering clinician. Thus, treating physicians’ only PET‐based indication of CAD would have been the presence of relative perfusion defects.

Death was assessed by clinical follow‐up and social security death index (SSDI) search.

### 2.1 Statistical analysis

All statistical analyses were performed using SPSS software, version 20 (SPSS Inc, Chicago, IL). Continuous variables are reported as mean ± SD, and categorical variables are given as n (%). Independent *t* tests were used to compare differences in means in continuous variables, and differences in proportions of categorical variables were evaluated with Fisher exact tests. Cox analysis, both univariable and multivariable, was used to assess relationships between risk factors and mortality, with survival displayed via the Kaplan‐Meier method and between‐group survival compared using the logrank test. For all tests, *P*  ≤ .05 was considered significant.

## 3 RESULTS

Among patients with AF who had undergone PET stress testing, 78 were taking a class 1C AAD at the time of PET scanning or within 1 month thereafter. Only one patient had started taking the 1C drug before undergoing PET stress (2 weeks prior). These patients were compared to the age‐matched control group of 78 patients with AF with no 1C AAD exposure.

As seen in Table 1, the groups were well‐matched on several factors, including age (1C AAD 68 ± 10 vs control 70 ± 9 years), LVEF (62% ± 4% vs 62% ± 5%), and whole‐heart sMBF (1.7 ± 0.7 vs 1.5 ± 0.6 cc/min/g). There were more males in the 1C group (68% vs 37%, *P* = .02). Among the control group, 11 (14%) were taking other AADs, including amiodarone (3 [4%]), dofetilide (3 [4%]), dronedarone (1 [1%]), and sotalol (4 [5%]). Beta blockers and calcium channel blockers were used by similar numbers of patients (69% vs 71%, *P* > .99 and 32% vs 40%, *P* = .4, respectively). We were able to calculate a valid Framingham risk score in 126 (81%; 61 controls and 66 1C patients). There was no significant difference in the Framingham risk between the two groups (12.6% vs 10.0%, *P* = .09). In addition, among 1C recipients, there was no significant difference in Framingham risk score upon stratification by the presence or absence of reduced CFC (10% vs 10%, *P* = .65).

**Table 1:** Baseline characteristics of the study population (n = 156)

	1C AAD group (n = 78)	Control group (n = 78)	*P* value
Age, y	68 ± 10	70 ± 9	.14
Sex, male	53 (68%)	37 (48%)	.02
LVEF	62 ± 4%	62 ± 5%	.69
Body mass index	36 ± 8	35 ± 8	.38
Systolic blood pressure	128 ± 22	132 ± 24	.24
Diastolic blood pressure	75 ± 13	73 ± 13	.30
Resting heart rate	67 ± 14	70 ± 15	.26
QRS duration	93 ± 13	105 ± 30	<.01
QTc	444 ± 28	455 ± 37	.04
Creatinine	1.0 ± 0.3	1.8 ± 2.2	<.01
Diabetes mellitus	21 (27%)	32 (41%)	.09
Hypertension	59 (76%)	64 (82%)	.43
Hyperlipidemia	40 (51%)	36 (46%)	.63
Left heart catheterization	5 (6%)	9 (12%)	.40
Echocardiographic regional wall motion abnormality	4 (5%)	9 (12%)	.25
Framingham risk score	12.6%	10.0%	.09
Whole heart sMBF, cc/min/g	1.7 ± 0.7	1.5 ± 0.6	.07
ACE/ARB	40 (51%)	49 (63%)	.20
Beta blocker	54 (69%)	55 (71%)	1.0
Calcium channel blocker	25 (32%)	31 (40%)	.40
Flecainide	59 (76%)	0 (0%)	N/A
Propafenone	19 (24%)	0 (0%)	N/A
Amiodarone	0 (0%)	3 (4%)	.25
Dofetilide	0 (0%)	3 (4%)	.25
Dronedarone	0 (0%)	1 (1%)	1.0
Sotalol	0 (0%)	4 (5%)	.12

Abbreviations: AAD, antiarrhythmic drug; ACE/ARB, angiotensin converting enzyme/angiotensin‐receptor blocker; LVEF, left ventricular ejection fraction; N/A, not applicable; sMBF, stress myocardial blood flow.

There were no patients in the control group and three patients in the 1C group with symptoms meeting the definition of typical angina as the indication for PET stress. None of the PET scans had perfusion abnormalities. During stress testing with dipyridamole, no patient experienced ST depressions consistent with ischemia, and one patient had chest pain at baseline that was unchanged during PET.

There were 13 patients (8%; six controls and seven 1C patients) who had stress testing in the year before PET stress. There were 14 patients (9%; nine controls and five 1C patients) who had previously undergone left heart catheterization, none of which showed any significant coronary occlusion.

The available follow‐up for the entire population was 719 ± 572 days. The 1C group was exposed to a 1C AAD for 604 ± 539 days, and the available follow‐up for controls was 835 ± 584 days. Among patients whose follow‐up time ended due to stopping the 1C drug, 6 patients stopped due to inefficacy, 1 due to cost, 9 due to drug intolerance, 14 following successful ablation of AF, and 5 patients adopted a rate control strategy for AF (rather than rhythm control). The end of follow up was reached due to death in three patients, and three patients were lost to follow up.

Sixteen control patients underwent cardiac catheterization (three for acute coronary syndrome), at a mean of 18 ± 24 months following PET. Seven of these patients underwent percutaneous coronary intervention, and one patient underwent coronary artery bypass grafting. Among the 1C group, three patients underwent catheterization at 135 to 1342 days following PET; two patients in preparation for transcatheter aortic valve replacement, and one for dyspnea. All three studies revealed nonobstructive CAD.

There were 19 total deaths: 7 in the 1C AAD group and 12 in the control group. Seventeen of these deaths were ascertained via clinical follow‐up and two deaths were found by SSDI search. In the 1C AAD group, there was one sudden cardiac death and no other cardiac deaths. In the control group, there was one sudden cardiac death and two nonsudden cardiac deaths. The one sudden death in the control group occurred in a patient with end‐stage renal disease. Following a missed dialysis treatment, ventricular tachycardia (VT) arrest, followed by PEA, resulted in death. The two nonsudden cardiac deaths in the control group occurred due to acute diastolic heart failure, one in the setting of urosepsis and non‐ST‐elevation MI, and the other in the setting of severe MR.

The one sudden cardiac death in the 1C group occurred in a patient with a history of emphysema and stage 3 lung cancer who was found unresponsive after being seen 5 minutes earlier in their usual state of health. The presenting rhythm was PEA and the patient was not revivable despite resuscitation efforts.

On Cox analysis, the 1C group and the matched control group had similar survival (hazard ratio [HR], 0.75; 95% confidence interval [CI], 0.29‐1.90; *P* = .54). Following multivariable correction for all significant (*P* ≤ .1) covariates (ie, QRS duration and creatinine), this relationship persisted (HR, 0.90; 95% CI, 0.32‐2.5; *P* = .83).

**Figure 1:**
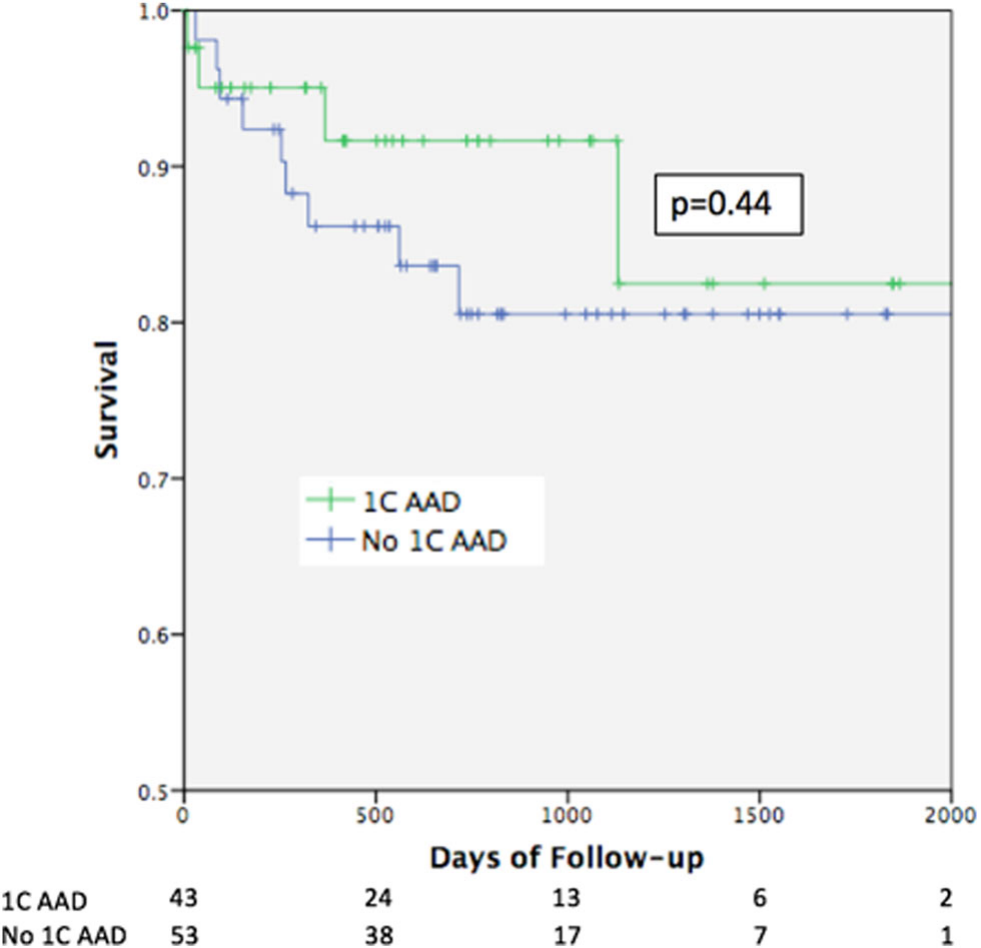
Kaplan‐Meier survival analysis of patients with greater than 50% of myocardium showing reduced CFC, stratified by 1C AAD use. AAD, antiarrhythmic drug; CFC, coronary flow capacity

Among patients whose PET‐derived CFC indicated the presence of CAD (ie, reduced CFC involving ≥50% of the myocardium: 43 [55%] 1C AAD patients and 53 [68%] controls), those treated with 1C AADs had survival similar to the matched control group not treated with 1C agents (HR, 0.63; *P* = .44). The two groups’ survival over time is depicted in the Kaplan‐Meier plot (Figure 1), with logrank survival analysis showing a nonsignificant difference between the curves (*P* = .44).

## 4 DISCUSSION

This study investigated the safety of 1C AAD use in patients with CFC indicating occult CAD. Among these patients, we found no difference in survival whether they were treated with a 1C AAD or not.

Since the initial publication of the CAST investigation in 1989, class 1C AADs have been considered unsafe for use in patients with CAD or other structural heart disease.^3^, ^10^ The CAST trial studied the use of 1C AADs in 1500 patients with a MI, a depressed LVEF, and frequent PVCs. The study found that 1C AADs were effective at suppressing PVCs, but the 1C group's significantly higher sudden death mortality was enough to halt the trial and restrict significantly the use of 1C AADs.^3^, ^10^ While these results were important, in common practice, the findings of the CAST trial have been generalized from the studied group (ie, patients with an MI and a depressed LVEF) to all patients with any CAD being treated as having increased risk if treated with 1C AAD, irrespective of LV function. While CAST showed that patients with a prior MI are at increased risk when taking 1C AADs, likely due to ventricular tachyarrhythmias potentiated by myocardial fibrosis that acts as an anatomical substrate for reentry, patients with CAD but no prior MI may not share the same risk profile.

Current societal guidelines for the treatment of AF recommend flecainide and propafenone as first‐line agents for rhythm control. According to the AHA/ACC/HRS practice guidelines, however, 1C AADs should be avoided or used with caution in patients with CAD, or in the presence of sinus node or atrioventricular node dysfunction, heart failure, atrial flutter, infranodal conduction disease, Brugada syndrome, or liver disease.^2^ Flecainide use is also cautioned against in patients with renal dysfunction, and propafenone use is relatively contraindicated in patients with asthma.^2^

1C drugs may be safe to use in a broader array of patients than previously thought. For example, Frankel et al recently examined the safety of 1C AADs for suppression of PVCs in patients with PVC‐induced cardiomyopathy. Their study found that 1C AADs effectively suppressed PVCs, leading to LVEF recovery in the majority. In addition, no adverse events were observed during the treatment.^11^ That analysis, and the results of the current investigation, support the argument for reassessing the safety of 1C AADs in patients without MI but with structural heart disease. Future studies examining the safety of these drugs may further narrow the contraindications to their use.

Our aim was to reanalyze the safety of these effective medications in patients with occult CAD based on PET‐CFC but no clinical diagnosis of CAD. This study excluded patients with clinically significant CAD (and certainly those with prior MI), and used PET‐derived CFC to identify patients with CAD but without clinical evidence of ischemia. Among patients with occult CAD, this study did not show increased mortality in the 1C AAD group. Of note, only one patient was taking a class 1C AAD before enrollment, and for only 2 weeks’ duration. This indicates an absence of significant survivor bias, which is a strength of the study.

Our data suggest that 1C AADs may be safer than previously thought in patients with CAD but without a history of MI or clinical ischemia. However, further study in a larger prospective cohort is required to confirm these findings.

## 5 LIMITATIONS

This was a retrospective evaluation of a modestly sized cohort of patients who were evaluated at a single tertiary care center. The relatively small size of the population limits the study's power. Larger studies will be required to confidently establish safety. However, we did not detect any evidence of harm related to 1C AAD use. Other than the omission of patients with CAD or depressed LVEF, the population was unselected, which could introduce variation. However, the cohorts were well‐matched on several relevant clinical factors. Finally, 14% of the control group were taking a non‐1C AAD, which may have led to selection bias due to those patients being “survivors” of antiarrhythmic therapy. We cannot exclude the possibility that some patients in the “control” group suffered some amount of increased mortality caused by their use of non‐1C AADs. However, if such an effect were truly present, it would further strengthen the argument in favor of 1C AAD use rather than other drugs. Lastly, we recognize that not all sudden death is arrhythmic, or even cardiac, in nature. In light of this, we included detail about the two deaths (one in each group) adjudicated as sudden. While the sudden death in the control group was certainly arrhythmic (observed VT), the etiology of the sudden unobserved death in the 1C group may or may not have been arrhythmic and/or cardiac.

## 6 CONCLUSION

In AF patients with preserved LV function and without clinically known CAD, but with PET‐CFC indicating the presence of occult CAD, treatment with a 1C AAD is not associated with increased mortality. Larger studies are required to more confidently establish the safety of these drugs in the setting of stable CAD.
